# A Tough Bridge to Cross: Myocardial Bridging Associated With Dehydration-Induced Ischemia in an Endurance Athlete

**DOI:** 10.7759/cureus.103494

**Published:** 2026-02-12

**Authors:** Serafino A LaGalbo, Anupama Rao, Sean P Swearingen

**Affiliations:** 1 Medicine, University of Wisconsin Hospital and Clinics, Madison, USA; 2 Cardiology, Rush University Medical Center, Chicago, USA

**Keywords:** acute myocardial injury, athlete’s heart, dehydration, myocardial bridging, shared decision making

## Abstract

Myocardial bridging is a term used to describe an anatomic anomaly where an epicardial artery courses under an overlying myocardial “bridge.” This condition has rarely been associated with exercise-induced ischemia or other cardiac events. Here, we describe a case of an endurance athlete who presented with evidence of acute myocardial injury after developing chest pain during participation in a marathon. Further evaluation revealed myocardial bridging involving the left anterior descending coronary artery (LAD) as the likely cause of her symptoms. In elite athletes, exercise-induced cardiac remodeling combined with the underlying pathophysiology of myocardial bridging puts them at an increased risk of experiencing a cardiac event and presents unique challenges in management and shared decision-making, as these patients often desire to continue athletic competition at a high level.

In this case, various strategies were explored to manage this condition in collaboration with the patient to accomplish this goal. Ultimately, medical management was utilized in conjunction with emphasizing adequate hydration while providing the patient with objective parameters to guide her endurance training. Through describing cases where myocardial bridges (MB) cause significant symptomatic manifestations such as this one, we can hope to better understand this condition’s pathophysiology and identify the most practical ways to approach its management.

## Introduction

Myocardial bridging is a congenital anomaly in which a segment of an epicardial artery takes an aberrant course into the myocardium, becoming “tunneled” beneath an overlying muscular “bridge” [1]. In most cases, myocardial bridges (MB) are considered an incidental and clinically silent finding. A review of the literature reports only about 35 cases of MB associated with cardiac events [2]. Despite this, their clinical significance continues to be debated. While MB can be symptomatic in all patient populations, athletes present a unique challenge, as their increased activity level and cardiac remodeling place them at higher risk for complications secondary to MB. In this case report, we present a case of a 46-year-old athlete who experienced symptoms and signs of myocardial injury from MB via an uncommon pathophysiology. 

## Case presentation

**Figure 1:**
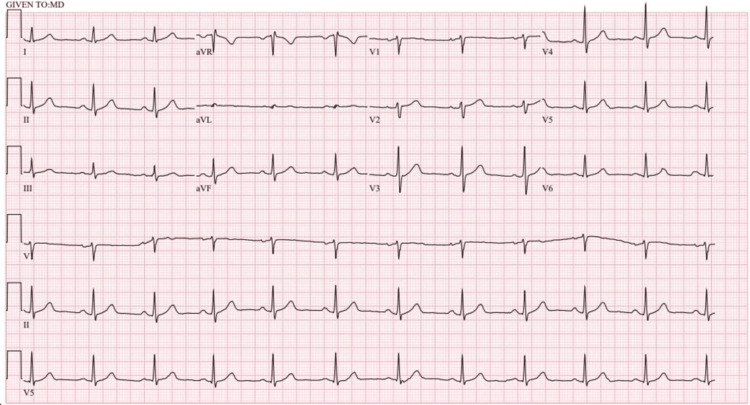
ECG demonstrating normal sinus rhythm without ischemic changes.

A 46-year-old woman with no pertinent medical history presented to the emergency department for chest pain that developed halfway through a marathon, causing her to stop the race. The initial physical exam was unremarkable, including stable vital signs (blood pressure 108/74 mmHg, heart rate 80 beats/min). An electrocardiogram (ECG) showed normal sinus rhythm and no acute ischemic changes (Figure 1). High-sensitivity troponin I was initially 33.7 ng/L (normal value < 17.0 ng/L), but on repeat, it had increased to 52.6 ng/L. Blood urea nitrogen (BUN) and creatinine were within normal limits. Her pain resolved, and her troponin trended down on a follow-up check after several liters of IV fluids and a baby aspirin provided by emergency medical services.

**Figure 2:**
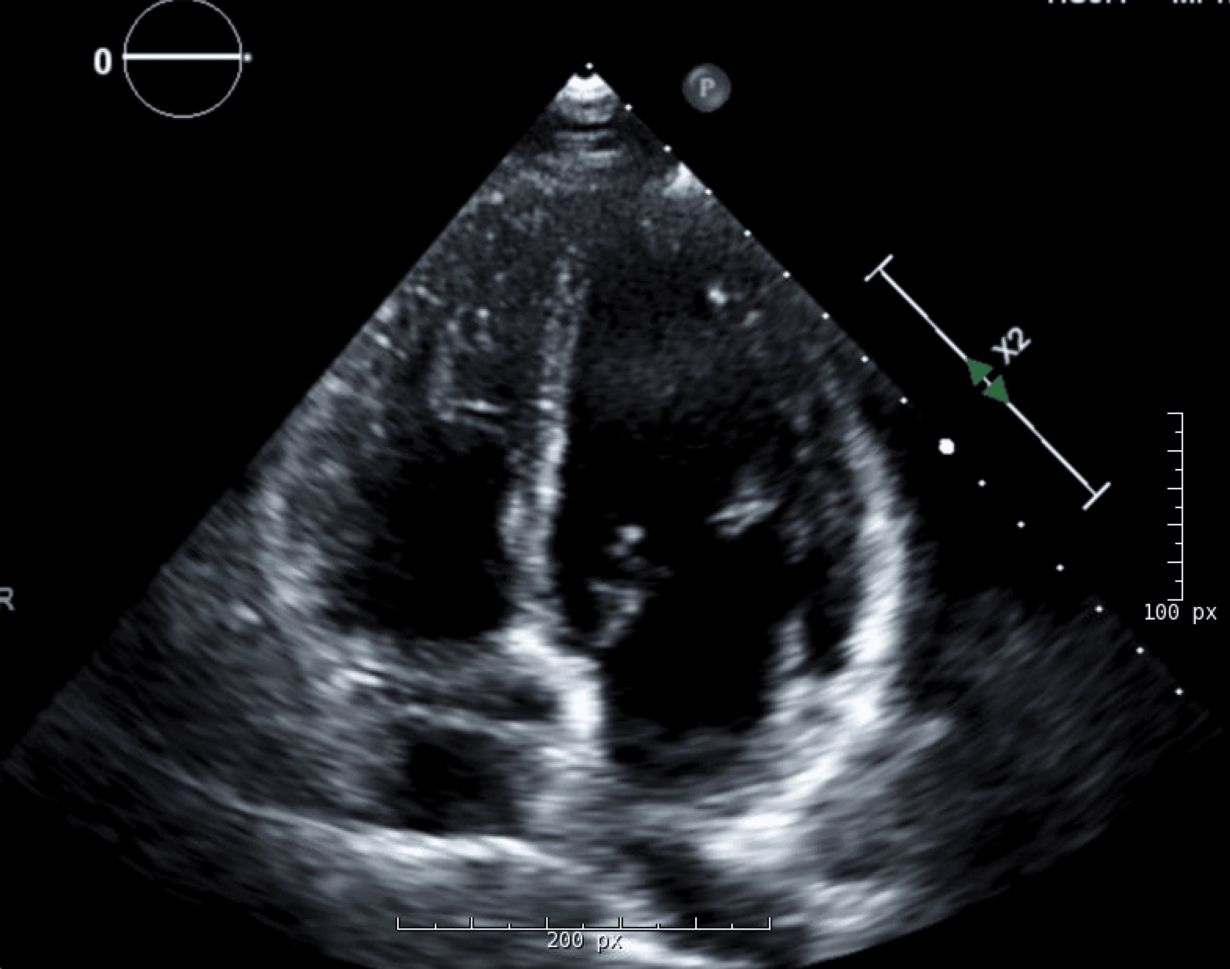
Transthoracic echocardiogram. Apical four-chamber view demonstrating appropriate right and left ventricular size and function.

**Figure 3:**
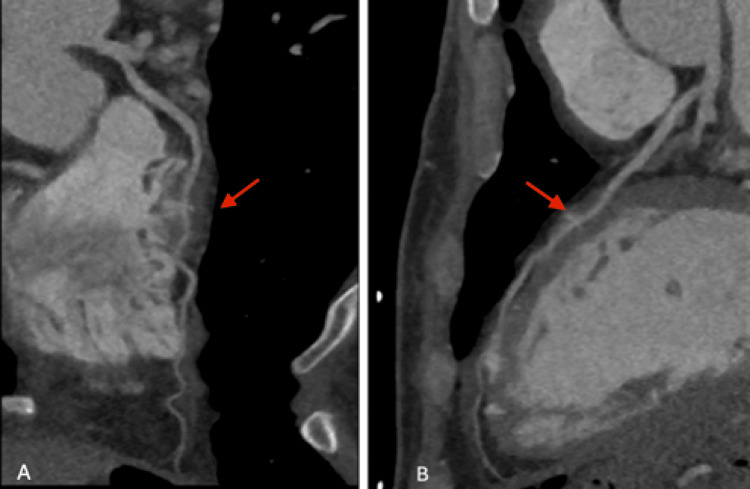
Coronary computed tomography angiography. A and B: Angiography showing myocardial bridging (arrow) of the left anterior descending coronary artery (LAD).

Serial ECGs remained normal, and the troponin assay began to downtrend after the initial increase. Echocardiography revealed normal ventricular and valvular function (Figure 2). Given the evidence of myocardial injury, but low risk for atherosclerotic plaque rupture, coronary computed tomography angiography (CCTA) was pursued and demonstrated normal origin of the right and left coronary arteries with no significant plaque, calcification, or stenosis. In the mid left anterior descending coronary artery (LAD), however, an MB was noted with a length of ~20 mm and a maximum depth of 3.6 mm (Figure 3).

**Figure 4:**
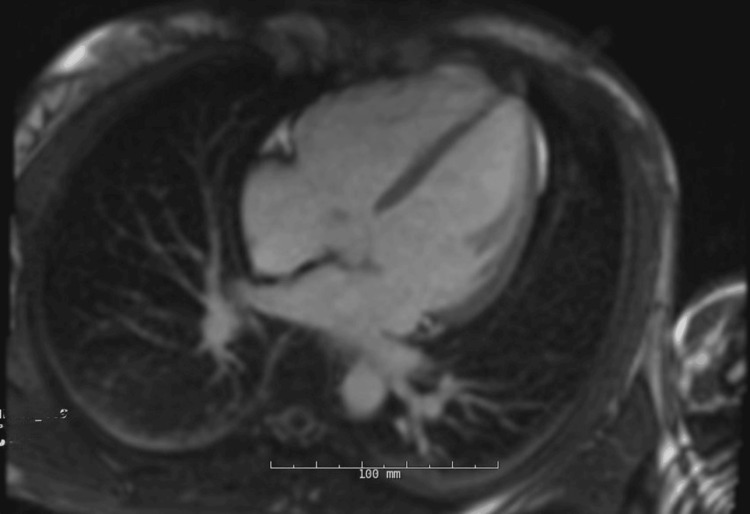
Cardiac magnetic resonance imaging. MRI demonstrating normal right and left ventricular size without evidence of fibrosis or scarring.

The patient was admitted and remained clinically and hemodynamically stable for 24 hours. A beta-blocker was initiated, and she was discharged with close follow-up and instructions to avoid intense exercise. Outpatient cardiac magnetic resonance imaging (MRI) demonstrated normal ventricular size and function and no evidence of scar or fibrosis (Figure 4).

The patient later reported shortness of breath and difficulty tolerating her normal level of exercise, which was ultimately attributed to her beta-blocker. These symptoms resolved after discontinuing the medication. She then underwent cardiopulmonary stress testing to gather more information about her cardiac function during rigorous exercise. She exercised for 15 minutes, achieving a maximum heart rate of 173 beats/min (99% of maximal predicted heart rate) and a maximal work rate of 17.5 METs. No ischemic changes were seen on stress ECG or echocardiogram, and her maximum oxygen consumption (VO2 max) was monitored throughout the test. Peak VO2 was 35 ml/kg/minute, and peak heart rate was 164 beats per minute. Heart rate at 75% of VO2 max was 145 beats per minute, and after a shared decision-making process, this was agreed upon to be her maximum target for exercise going forward. Adequate hydration was emphasized to avoid dehydration-related hemodynamic changes. Since her initial presentation, the patient has not reported any recurrence of her symptoms and continues to exercise by running at a moderate pace for three to four miles routinely.

## Discussion

MBs are most commonly found in the LAD, typically with a depth of 1 to 10 mm and a tunneled length of 10 to 30 mm [1]. The prevalence of MB is difficult to define, as different diagnostic modalities have produced variable results. In the modern era, CCTA provides a non-invasive and sensitive way to visualize the coronary arteries with high spatial resolution. This has made it the preferred method of diagnosing MB and has improved detection by about 10 times in comparison to the classically utilized coronary angiogram [3].

Generally, MBs are clinically silent; however, they can cause potentially lethal cardiac problems in a small subset of patients, particularly when the bridged vessels are deeper than 2 mm [4]. Numerous mechanisms have been proposed to cause these underlying pathologies, but among them, hemodynamic changes resulting in reduced coronary flow reserve seem to play a critical role [3]. This is made even more challenging in the context of athletes, who have a uniquely increased risk, as the inherent myocardial hypertrophy caused by exercise-induced cardiac remodeling may worsen the underlying pathophysiology of MB. Furthermore, increased inotropy and tachycardia secondary to exercise can further aggravate pathophysiologic disturbances in coronary flow. 

Augmenting coronary blood flow and avoiding symptomatic triggers are typically mainstays of treatment for symptomatic MB. Pharmacotherapy includes beta-blockers or calcium channel blockers due to their negative ionotropic effects and subsequent increase in coronary filling times [1]. Restriction of athletic activities is typically only recommended when there are significant symptoms or signs of infarct or ischemia, as was seen in this patient [5]. For these cases, guidelines suggest the use of an established classification system to limit patients to “low or moderate dynamic and static demands” [6]. “Moderate” dynamic activity can be defined as 75% of a patient’s VO2 max, which is able to be identified with a cardiopulmonary exercise stress test. “Moderate” static activity involves achieving up to 20% of the maximum muscle contraction, which is usually a subjective parameter. Using these definitions and cardiopulmonary testing can help identify a target heart rate that can be used as a benchmark to identify a specific level of activity. Providing the patient with these parameters allows them to continue exercising safely while mitigating risk. If medical intervention fails to control symptoms, a surgical approach, e.g., myotomy, may be beneficial in athletes who wish to return to full participation.

Interestingly, through further discussion during outpatient follow-up, dehydration was identified as a major factor in provoking this patient’s symptoms. Although her BUN and creatinine were not significantly elevated at the time of her presentation, the context of her presentation, following the marathon and improvement with symptoms and troponin with IV fluids, makes relative dehydration a major contributing factor to her presentation. While increased heart rate and cardiac contractility are typically recognized as the primary causes for MB-induced symptoms during exercise, an association with dehydration has also been explored as a potential provoking factor. This phenomenon was specifically seen in our patient as her cardiopulmonary exercise testing (CPET) took her to her maximum heart rate, but did cause ischemia as the patient was properly hydrated at the time and was not exercising for a prolonged time in warm weather. Maaliki et al. reported a case of a 50-year-old man presenting with syncope, hypotension, and ST elevation with angiographic confirmation of LAD bridging that resolved with aggressive IV hydration [7]. Normal physiologic responses to dehydration include increased viscosity and decreased blood volume return to the heart, both of which result in decreased filling pressure and stroke volume while increasing heart rate. This indicates that a focus on maintaining hydration throughout physical exertion could be a viable strategy for mitigating MB-driven symptoms.

Ultimately, management of MB in athletes should focus on minimizing symptoms and cardiovascular risk with shared decision-making regarding what physical activities are considered to have an acceptable risk. This cooperative approach to management can be seen in a case described by Alexandre et al. that involved the sudden collapse of a marathon runner who was found to have three simultaneous MBs. In this case, the patient suffered significant cardiac symptoms and hemodynamic compromise, resulting in cardiopulmonary resuscitation (CPR) in the field. Work-up revealed the etiology to be extensive MBs, and subsequent discussions were held in regard to the best strategy for management. Options including coronary artery bypass grafting (CABG), myotomy, and conservative management were discussed, and the patient ultimately opted for conservative management and refrained from further competitive sports and marathon running [8]. While similar discussions around management were held in our case, the severity of the symptoms and desire of the patient to continue to compete were taken into consideration and caused management to differ.

## Conclusions

Despite MB usually being a silent incidental finding, they can be particularly challenging to manage in symptomatic patients and could have serious complications. Athletes prove to be some of the most challenging patients in which to manage symptomatic MB due to the physiologic changes the heart undergoes during rigorous exercise, thus affecting the pathophysiology of this condition. Several strategies can be used to manage MB with varying levels of invasiveness and lifestyle restrictions. This makes collaborative decision-making with the patient’s goals in mind essential to successful management. Through continuing to document cases of this condition in particularly challenging populations, we can hope to better guide management strategies in a more patient-centered fashion.

## References

[1] Lee MS, Chen CH (2015). Myocardial bridging: an up-to-date review. J Invasive Cardiol.

[2] Gowd BM, Thompson PD (2014). Isolated myocardial bridging and exercise-related cardiac events. Int J Sports Med.

[3] Tarantini G, Migliore F, Cademartiri F, Fraccaro C, Iliceto S (2016). Left anterior descending artery myocardial bridging: a clinical approach. J Am Coll Cardiol.

[4] Santucci A, Jacoangeli F, Cavallini S, d'Ammando M, de Angelis F, Cavallini C (2022). The myocardial bridge: incidence, diagnosis, and prognosis of a pathology of uncertain clinical significance. Eur Heart J Suppl.

[5] Thompson PD, Myerburg RJ, Levine BD, Udelson JE, Kovacs RJ (2015). Eligibility and disqualification recommendations for competitive athletes with cardiovascular abnormalities: Task Force 8: coronary artery disease: a scientific statement from the American Heart Association and American College of Cardiology. J Am Coll Cardiol.

[6] Levine BD, Baggish AL, Kovacs RJ, Link MS, Maron MS, Mitchell JH (2015). Eligibility and disqualification recommendations for competitive athletes with cardiovascular abnormalities: Task Force 1: classification of sports: dynamic, static, and impact: a scientific statement from the American Heart Association and American College of Cardiology. J Am Coll Cardiol.

[7] Maaliki N, Omar M, Ali AA, Roemer A, Ruiz J, Sadic E (2021). Myocardial bridging unmasks as an acute coronary syndrome from dehydration. Case Rep Cardiol.

[8] Alexandre A, Vieira P, Dias-Frias A (2022). Myocardial bridging leading to cardiac collapse in a marathon runner. J Cardiovasc Dev Dis.

